# Novel Drug–Drug Cocrystalline Forms of Carbamazepine with Sulfacetamide: Preparation, Characterization, and In Vitro/In Vivo Performance Evaluation

**DOI:** 10.3390/pharmaceutics17050678

**Published:** 2025-05-21

**Authors:** Denis E. Boycov, Ksenia V. Drozd, Alex N. Manin, Andrei V. Churakov, Mikhail Yu. Vlasov, Irina V. Kachalkina, German L. Perlovich

**Affiliations:** 1 G.A. Krestov Institute of Solution Chemistry of the Russian Academy of Sciences, 1 Akademicheskaya St., Ivanovo 153045, Russia; denboycov11@gmail.com (D.E.B.); ksdrozd@yandex.ru (K.V.D.); anm@isc-ras.ru (A.N.M.); 2 Institute of General and Inorganic Chemistry of the Russian Academy of Sciences, 31 Leninsky Prosp., Moscow 119991, Russia; churakov@igic.ras.ru; 3 Research Institute of Biotechnology “BioTech”, Samara State Medical University of the Ministry of Health of the Russian Federation, 89 Chapayevskaya St., Samara 443099, Russia; m.yu.vlasov@samsmu.ru (M.Y.V.); i.v.sokolova@samsmu.ru (I.V.K.)

**Keywords:** carbamazepine, sulfacetamide, cocrystal, crystal structure, phase diagram, stability, dissolution, permeability, pharmacokinetics

## Abstract

**Objectives**: Drug–drug cocrystallization represents a promising approach for the development of novel combination drugs with improved physicochemical and biopharmaceutical properties. The aim of the present research is to prepare novel drug-drug cocrystalline forms of antiepileptic drug carbamazepine (CBZ) with sulfacetamide (SCTM). **Methods**: The novel CBZ cocrystal methanol solvate and cocrystal hydrate were prepared via solvent evaporation technique and characterized by single crystal X-ray diffraction, differential scanning calorimetry and thermogravimetric analysis. **Results**: Single-crystal X-ray diffraction and thermal analysis revealed that the multicomponent solids are isostructural, wherein the solvent molecule does not play a structure-forming role. To optimize the synthesis of [CBZ+SCTM+H_2_O] (1:1:0.7), the binary and ternary phase diagrams were constructed in acetonitrile at 25 °C. A thorough investigation of the cocrystal hydrate behavior in aqueous solution showed that the pH of the dissolution medium exerted a significant effect on the stability and solubility of [CBZ+SCTM+H_2_O] (1:1:0.7). According to the dissolution and diffusion experiments in a buffer solution pH 6.5, the cocrystal hydrate characterized an enhanced dissolution rate and flux of CBZ. Pharmacokinetic studies in rabbits showed that the novel cocrystal hydrate exhibited a comparable bioavailability to the parent CBZ. **Conclusions**: Overall, this work reports the preparation of a novel CBZ drug-drug cocrystal hydrate, which can be considered as an alternative CBZ solid form for oral usage, possessing additive pharmacological effect.

## 1. Introduction

The creation of alternative solid forms of active pharmaceutical ingredients (APIs) by introducing an additional component, which modifies intermolecular interactions in the underlying crystal structure, represents a promising approach for fine-tuning of physicochemical and biopharmaceutical properties [1,2,3,4,5,6,7,8]. In recent years, considerable advances have been witnessed in the realm of virtual screening tools for multicomponent crystals [9,10,11,12,13]. However, the inherent limitations and challenges of prediction tools continue to preclude the possibility of excluding the utilization of experimental screening for the identification of novel solid forms of drug substances [14].

Currently, solution-based methods constitute a significant part of the experimental techniques employed for screening and synthesis of multicomponent crystals [15]. In these methods, the solution assumes a pivotal role, and its variation enables the control of the distinct characteristics of the solids [16,17,18,19]. In certain cases, the synthesis of multicomponent crystals using solution-based methods requires accurate knowledge of phase diagram. Examples of this may include instances where the solubilities of the parent components differ significantly, or where the concurrent formation of multiple solid phases of different composition is feasible [20,21,22,23]. It should be noted that the primary risk associated with the utilization of solvents is their capacity to be incorporated into the crystal lattice of multicomponent crystals [24,25,26,27]. From a pharmaceutical perspective, the majority of organic solvents are known to have toxic and carcinogenic effects on the human body, and their presence in the final product must be completely eliminated [28,29]. In contrast, the safety of water for humans makes the use of hydrated forms of pharmaceutical substances acceptable and widely available [30].

**Figure 1:**
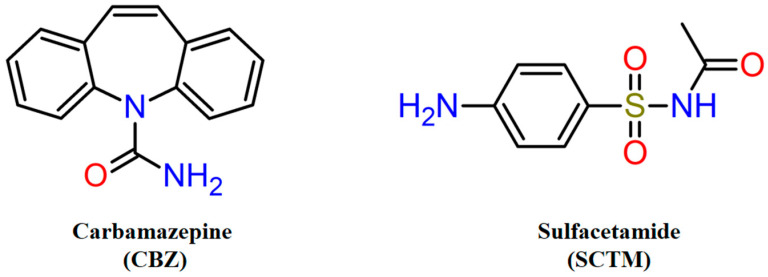
Chemical structures of active pharmaceutical ingredients applied for cocrystallization.

Carbamazepine (CBZ, Figure 1) is a dibenzoazepine derivative, possessing antiepileptic, neurotropic, and psychotropic activity [31,32]. Despite CBZ’s long history of use since 1963, it remains a significant pharmaceutical agent in treatment of epilepsy [33]. According to the Biopharmaceutical Classification System (BCS), CBZ belongs to BCS class II [34], and its dissolution exerts a rate-limiting effect on oral absorption and hence on the bioavailability of the drug [35]. In recent decades, CBZ has been the subject of extensive study, with a range of approaches being evaluated for enhancing its solubility and dissolution rate in water, such as preparation of solid dispersions [36,37,38,39], twin-screw melt granulation [40], complexation with cyclodextrins [41,42], micronization [43], nanoslurry [44], co-amorphization [45], cocrystallization [46,47,48], and others. Among the aforementioned approaches, cocrystallization has received particular attention, as evidenced by the number and diversity of CBZ cocrystals [49]. A special subset of the CBZ multicomponent crystals is constituted by drug–drug cocrystals due to their additive or synergistic pharmacological effects [50]. A survey of the Cambridge Structural Database (CSD) and scientific literature revealed that CBZ was cocrystallized with a range of pharmaceuticals [49], including non-steroidal anti-inflammatory drugs [51,52,53,54], antibiotic [27], antimalarial [55], diuretic [56], anticancer [57], and anti-inflammatory [58] drugs.

In the present work, we perform a novel drug–drug cocrystal of CBZ with sulfacetamide (SCTM, Figure 1). SCTM is a constituent of the sulfonamide antibiotic class, exhibiting antibacterial [59] and anti-inflammatory activity, as well as an ability to suppress neuropathic pain [60]. While the solubility of SCTM in water is limited [61,62], it is nevertheless superior to that of CBZ, thus giving rise to the prospect that the cocrystallization of these APIs could lead to enhanced dissolution behavior for CBZ. In the course of the screening and synthesis of a novel multicomponent crystal based on CBZ and SCTM, it was determined that the cocrystal is prone to forming hydrated and solvated forms. In view of the potential risks associated with the use of methanol-solvated cocrystals in pharmaceutical formulations, a series of in vitro and in vivo studies, including dissolution, diffusion, and pharmacokinetics, was performed only for the hydrated cocrystal of CBZ and SCTM.

## 2. Materials and Methods

### 2.1. Materials

Polymorphic form III of CBZ (C_15_H_12_N_2_O, 99.42%) was purchased from BLD Pharm (Shanghai, China), SCTM (C_8_H_10_N_2_O_3_S, >98%), and hydroxypropyl methylcellulose (HPMC, average molecular weight is ~10,000) were sourced from Sigma-Aldrich (St. Louis, MO, USA). Acetonitrile (ACN) and methanol (MeOH) of HPLC-grade were used as mobile phases and/or solvents for screening, crystallization, and preparation procedures. The remaining organic solvents used in this study were of analytical grade purity.

### 2.2. Screening and Preparation of Solid Phases

#### 2.2.1. Grinding Method

Multicomponent crystal screening was carried out in a Planetary Micro Mill, model Pulverisette 7 (Fritsch, Idar-Oberstein, Germany). Prior to each experiment, a total amount of 50 mg of the (CBZ+SCTM) physical mixture in a 1:1 molar ratio was carefully weighed and placed in a 12 mL agate grinding jar, with twelve agate balls with a diameter of 5 mm included. Further, 50 μL of the appropriate solvent was added to the physical mixture using a micropipette. The grinding procedure consisted of two 30 min grinding cycles, with a 5 min break between them to prevent overheating of the samples. The rotation speed was set at 500 rpm. Following the grinding process, the open jars were left for several hours at ambient temperature in order to facilitate the evaporation of the residual solvent. The resulting powders were analyzed using PXRD by comparing the experimental PXRD patterns to each other and to the parent components.

#### 2.2.2. Solution Crystallization

The single crystals of the cocrystal hydrate ([CBZ+SCTM+H_2_O] (1:1:0.7)) and cocrystal methanol solvate ([CBZ+SCTM+MeOH] (1:1:0.352)) were harvested from saturated acetonitrile and methanol solutions, respectively. The crystallization of both solids was achieved through the dissolution of 50–60 mg of the (CBZ+SCTM) physical mixture in a 1:2 molar ratio in 1 mL of a solvent within a test tube at room temperature. Each test tube was hermetically sealed using Parafilm, which was punctured with three holes to facilitate slow evaporation of the solvent. After a period of 2–3 days, the single crystals with similar morphology were observed on the walls of each test tube. The crystals were then gently extracted from the solution and dried under ambient conditions for further investigation.

#### 2.2.3. Slurry

The slurry technique was utilized to facilitate the large-scale production of [CBZ+SCTM+H_2_O] (1:1:0.7) in powder form. The procedure involved the simultaneous mixing of 190 mg of CBZ, 210 mg of SCTM, and 763 μL of ACN using a magnetic stirrer at 300 rpm over a period of 3 days at room temperature. Subsequent to this, the solution was immediately separated from the solid phase via vacuum filtration to prevent crystallization of the parent components. The resultant powder was then dried under ambient conditions and analyzed by PXRD.

### 2.3. Powder X-Ray Diffraction (PXRD)

The PXRD analysis was conducted using Bragg–Brentano geometry on a D2 Phaser diffractometer equipped with a LYNXEYE XE-T detector (Bruker AXS, Karlsruhe, Germany). The generation of X-ray radiation was produced by a Cu anode source (*λ*_CuKα1_ = 1.5406 Å), operating at 30 kV voltage and 10 mA current. The experimental data were collected in a 5–30° 2θ range at a scanning rate of 0.02°.

### 2.4. Single-Crystal X-Ray Diffraction (SCXRD)

The crystal structures of CBZ cocrystal hydrate and methanol solvate were determined using SMART Photon II and D8 Venture diffractometers (Bruker AXS, Karlsruhe, Germany), respectively, both equipped with Mo-*K*α radiation sources (*λ* = 0.71073 Å, graphite monochromator). Absorption correction was applied by SADABS [63]. The crystal structures were solved by direct methods and refined by full-matrix least-squares on *F*^2^ with anisotropic thermal parameters for all of the non-hydrogen atoms [64]. All hydrogen atoms were found from difference Fourier synthesis and refined isotropically (except for the disordered phenyl ring in the methanol solvate). In the hydrate structure, disordered water molecules were removed from the model by the SQUEEZE procedure [65]. The CIF files are available from the Cambridge Crystallographic Data Centre under the CCDC numbers 2442630 and 2442631. This information is free of charge and can be obtained from the Cambridge Crystallographic Data Centre at www.ccdc.cam.ac.uk/structures (accessed on 19 May 2025).

### 2.5. Thermal Analysis

#### 2.5.1. Differential Scanning Calorimetry (DSC)

The thermal behavior of the studied solids was investigated by means of a DSC 4000 (PerkinElmer, Waltham, MA, USA) with a refrigerated cooling system. Prior to analysis, the DSC apparatus was meticulously calibrated using indium and zinc standard samples. In each measurement, the powder (2–3 mg) was weighed in an aluminum crucible and heated at a rate of 10 °C·min^−1^ with a temperature range from 25 to 200 °C. The inert atmosphere within the DSC apparatus was maintained through the implementation of a 20 mL·min^−1^ flow rate of dry nitrogen.

#### 2.5.2. Thermogravimetric Analysis (TGA)

The desolvation behavior of the multicomponent crystals was investigated via a TG 209 F1 Iris thermomicrobalance (Netzsch, Selb, Germany). The quantity of the bulk sample, ranging from 4 to 6 mg, was placed in a platinum pan and subsequently heated at a 10 °C·min^−1^ rate within a temperature range from 25 to 200 °C. The inert atmosphere within the TGA apparatus was maintained through the implementation of a 30 mL·min^−1^ flow rate of dry argon.

### 2.6. High-Performance Liquid Chromatography (HPLC)

The concentrations of CBZ and SCTM were measured using the LC-20 AD Shimadzu Prominence liquid chromatograph, equipped with a PDA detector and a Luna C-18 column (150 mm × 4.6 mm i.d., 5 μm particle size and 100 Å pore size). The temperature of the HPLC column was set to 40 °C. To achieve CBZ and SCTM separation on the column, a mobile phase consisting of ACN and a 0.1% aqueous solution of trifluoroacetic acid in a 40/60 volume ratio was utilized. The detection of CBZ and SCTM was performed based on UV-Vis absorption at wavelengths of 284 nm and 272 nm, respectively.

### 2.7. Determination of the Binary and Ternary Phase Diagrams

The phase diagrams for the CBZ-SCTM system in acetonitrile were determined by adding excess CBZ or SCTM to different concentrations of solutions of SCTM or CBZ. The resulting suspensions were shaken in a ThermoMixer C instrument (Eppendorf, Hamburg, Germany) at 800 rpm for 72 h at 25 °C. After equilibration, the suspension was withdrawn by syringe and filtered through a 13 mm hydrophilic PTFE filter with a pore size of 0.22 μm. Subsequently, an aliquot of the filtrate was taken with a micropipette and diluted with the mobile phase. The concentrations of the components in the diluted solutions were measured using an HPLC method. The residual phases after all solubility tests were collected and evaluated by PXRD. Each experimental point was replicated thrice.

### 2.8. In Vitro Studies

#### 2.8.1. Solubility Experiments

The solubility-pH profile of the parent SCTM was determined by measuring its equilibrium solubility in buffer solutions with pH 3.6, 6.5, and 7.4 at 37 °C. In each solubility test, an excess of SCTM was added to 2 mL of buffer solution, and the suspension was equilibrated at 37 °C for 72 h. Then, the suspension was filtered through a PTFE filter with a pore size of 0.22 μm, and the saturated solution was diluted with a mobile phase. The SCTM concentration was measured by the HPLC method. The pH of the solution was re-measured immediately after filtration.

The thermodynamic stability and solubility of [CBZ+SCTM+H_2_O] (1:1:0.7) were evaluated at the eutectic point in buffer solutions with pH 3.6 and 6.5 at 37.0 °C. In order to reach the eutectic point, an excess of CBZ dihydrate (30 mg) and cocrystal hydrate (100 ÷ 200 mg) was equilibrated in 2 mL of buffer solution at 37 °C for 72 h. After equilibration, the sample was filtered, and the CBZ and SCTM concentrations were measured by the HPLC method after dilution with a mobile phase. The phase composition of the residual solids after drying at room temperature was controlled by PXRD. Each experiment was replicated thrice.

#### 2.8.2. Powder Dissolution Under Non-Sink Conditions

The dissolution behavior of [CBZ+SCTM+H_2_O] (1:1:0.7) in aqueous media was studied using a USP-certified EDT-08LX dissolution tester (Electrolab, Mumbai, India). For each experiment, 470 mg of the cocrystal hydrate was added to a dissolution jar. Subsequently, the jar was filled with 300 mL of the pre-heated (at 37 °C) phosphate buffer solution pH 6.5 (blank FaSSIF), both in the absence and presence of pre-dissolved HPMC (0.1% *w*/*v*). The resulting suspension was continuously stirred by the paddle at 75 rpm for 360 min. Throughout the experiment, the temperature inside the jar was maintained at 37 °C using a water bath. At pre-determined time intervals, such as 5, 10, 15, 20, 30, 45, 60, 90, 120, 180, 240, 300, and 360 min, an aliquot of suspension of 1 mL was extracted from the jar and filtered through the 13 mm hydrophilic PTFE syringe filter with a pore size of 0.22 μm. In order to maintain a constant volume of dissolution medium, the withdrawn volume of suspension was compensated for on each occasion with an appropriate fresh buffer solution. The filtered aqueous solution was then diluted with the mobile phase, and the concentrations of both drugs were measured using the HPLC method. At the end of the dissolution experiment, after 6 h of each dissolution test, the pH of the aqueous medium was re-measured. The residual solid was extracted, dried at ambient conditions, and analyzed by PXRD. Each dissolution test was replicated thrice.

#### 2.8.3. Diffusion Tests

The diffusion behavior of the CBZ cocrystal hydrate was evaluated using an H1C side-by-side diffusion system (PermeGear, Hellertown, PA, USA) and a biomimetic lipophilic barrier PermeaPad^®^ (PHABIOC GmbH, Karlsruhe, Germany). The volume of the donor and receptor chambers was 7 mL. An effective permeation area of the membrane was 1.77 cm^2^. For each experiment, 11 mg of the cocrystal hydrate was placed in the donor chamber. Then, each chamber was simultaneously filled with 7 mL of the blank FaSSIF, both in the absence and presence of pre-dissolved HPMC (0.1% *w*/*v*). The contents of each chamber were stirred by a magnetic stir bar at 500 rpm. Throughout the experiment, the temperature inside the chambers was maintained at 37 °C by circulating water within a heating jacket. At pre-determined time intervals, such as 30, 60, 120, 180, 240, 300, and 360 min, an aliquot of 400 μL was extracted from the receptor chamber using a micropipette. Subsequent to the withdrawal of each aliquot, the volume was replenished with the appropriate fresh buffer solution. The concentration of the diffused CBZ through the biomimetic lipophilic membrane was measured by HPLC.

### 2.9. In Vivo Pharmacokinetics

The rabbits of Soviet chinchilla bread (4.53 ± 0.07 kg) were used to perform a pharmacokinetic investigation of the novel cocrystal hydrate. Prior to the in vivo tests, the animals were held under quarantine with unlimited access to food and water for 3 weeks. For the 12 h before the in vivo tests, the animals were deprived of food but had free access to water. Subsequently, the gelatin capsules containing 60 mg·kg^−1^ of CBZ or an equivalent amount of cocrystal hydrate were orally administered to rabbits using a tablet introducer. After capsule administration, the blood samples were collected from the marginal ear vein at the following time intervals: 0.5, 1, 2, 3, 4, 6, 9, 12, 24, and 48 h. The blood samples were centrifuged at 3000 rpm for 15 min. Plasma was collected in Eppendorf tubes and stored at −80 °C. Subsequently, the 0.5 mL of plasma was diluted with ACN and the solution was vortexed and centrifuged at 10,000 rpm for 10 min. The CBZ concentration in the samples was measured by HPLC.

## 3. Results and Discussion

### 3.1. Effect of Solvents on Cocrystallization of CBZ and SCTM by Grinding

**Figure 2:**
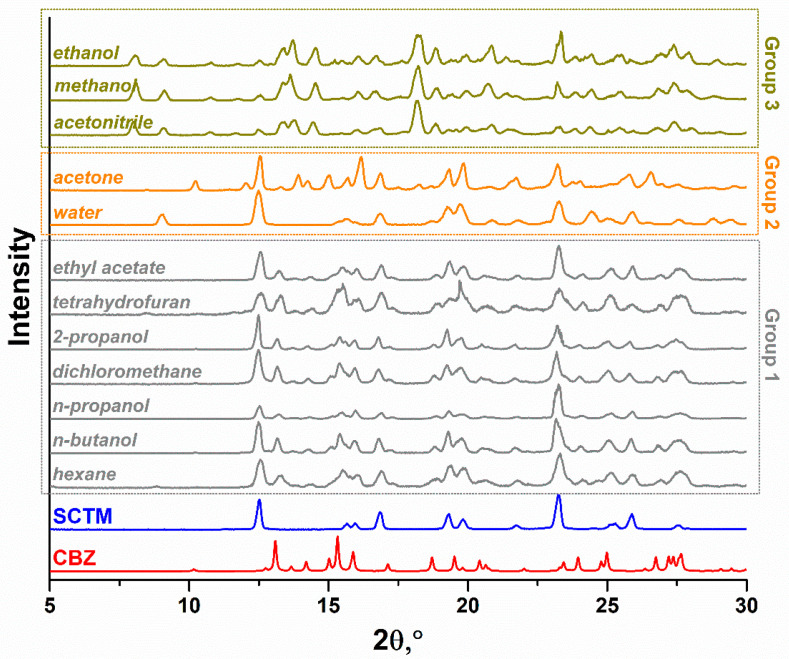
Experimental PXRD patterns of the ground (CBZ+SCTM) physical mixtures (1:1) with different solvents. The groups of PXRD patterns identify the nature of LAG product: a physical mixture of parent drugs (Group 1, grey), a mixture of CBZ solvate and SCTM (Group 2, orange), or a novel multicomponent solid between CBZ and SCTM (Group 3, dark yellow).

Extensive experience demonstrates that liquid-assisted grinding (LAG) is one of the most prevalent techniques for the screening and synthesis of the CBZ multicomponent crystals, such as binary and higher-order cocrystals [27,66,67], cocrystal hydrates/solvates [68,69], and polymorphs [70]. Concurrently, the outcome of the CBZ cocrystal synthesis by LAG can be considerably influenced by the nature of the selected solvent [71]. Therefore, in order to identify potential multicomponent solid forms between CBZ and SCTM, a series of 12 different solvents were tested in the LAG experiments, including hexane, n-butanol, n-propanol, dichloromethane, 2-propanol, tetrahydrofuran, ethyl acetate, water, acetone, acetonitrile, methanol, and ethanol. The experimental PXRD patterns of the obtained samples are shown in Figure 2.

As demonstrated in Figure 2, all of the experimental PXRD patterns can be divided into three distinct groups, thereby confirming that even a minimal amount of solvent utilized during the LAG experiments can exert a significant impact on the final solid. Group 1 (grey) comprises 7 PXRD patterns, wherein all reflections exhibit a complete correspondence to the superposition of the characteristic peaks of the parent components. This indicates that the grinding of CBZ with SCTM in the presence of hexane, n-butanol, n-propanol, dichloromethane, 2-propanol, tetrahydrofuran, or ethyl acetate does not result in the formation of the novel crystalline solid. The PXRD patterns obtained during the grinding of (CBZ+SCTM) by the addition of water or acetone were united in Group 2 (orange). Despite the evident disparities between the experimental PXRD patterns, they are converging at a comparable point in the transition of the parent CBZ during the grinding process in these solvents. According to CSD analysis, CBZ is known to exist in dihydrate form and 17 solvates [49], including the acetone solvate [72]. A comparison of the PXRD patterns from Group 2 with the calculated PXRD patterns for CBZ dihydrate (CBZ·2H_2_O) or acetone solvate was performed (Figure S1). It was determined that all the peaks differing from those exhibited by the parent SCTM are concomitant with those observed in these forms of CBZ. This finding suggests that the use of water or acetone in the grinding of the parent components, as observed in Group 1, does not lead to the formation of a new multicomponent solid between CBZ and SCTM. The final Group 3 (dark yellow) includes a set of comparable PXRD patterns that were obtained via the grinding of CBZ and SCTM in the presence of acetonitrile, methanol, or ethanol. The PXRD patterns exhibited by this group deviated considerably from those observed in the other two groups, as well as from the patterns of the parent components. Moreover, the absence of data concerning the formation of CBZ or SCTM of solvates with these particular organic solvents may indirectly signify the formation of new multicomponent solid or solids. In order to guarantee this, the experimental PXRD patterns from Group 3 were additionally compared with the calculated PXRD patterns of the CBZ polymorphs to exclude the possibility of its polymorphic transition during LAG (Figure S2). Consequently, subsequent to the elimination of all possible transformations of initial components during the grinding process using acetonitrile, methanol, and ethanol, a conclusion can be reached that the obtained unique PXRD patterns indicate precisely the formation of a new multicomponent product.

### 3.2. Crystal Structure Analysis

In order to ascertain the reason for the obtaining of identical PXRD patterns when the (CBZ+SCTM) physical mixture was ground in the presence of acetonitrile, methanol, and ethanol, the same organic solvents were used for crystallization experiments. Preliminary experiments revealed that, due to different solubilities of the parent components in the selected solvents, the use of the equimolar physical mixtures resulted in the concurrent crystallization of needle-like single crystals of CBZ form III and powder of a novel multicomponent solid. On this basis, a series of crystallization experiments were performed for the (CBZ+SCTM) physical mixtures with a twofold excess of SCTM. In consequence of the crystallization from acetonitrile and methanol, the single crystals were obtained. The SCXRD analysis revealed that these crystals corresponded to a cocrystal hydrate ([CBZ+SCTM+H_2_O] (1:1:0.7)) and a cocrystal methanol solvate ([CBZ+SCTM+MeOH] (1:1:0.352)), respectively. The crystallographic data for the novel CBZ multicomponent crystals are collected in Table 1. A comparison of the calculated PXRD patterns for the novel solids with each other showed a high degree of similarity, with only minor discrepancies observed at high 2θ angles (Figure S3).

It is evident that both multicomponent crystals belong to the same *P*-1 space group of the triclinic system. The asymmetric units of these crystals contain one molecule of CBZ and SCTM, in addition to a solvent molecule displaying different site occupancy. The occupancy factor of the methanol molecule in [CBZ+SCTM+MeOH] was found to be 0.352. Conversely, the water molecule in [CBZ+SCTM+H_2_O] exhibited full disorder and was removed via the SQUEEZE procedure. Therefore, the water content in the crystal structure was determined via TG analysis. It should be noted that the incorporation of water into the crystal lattice of the CBZ multicomponent crystals when using organic solvents is a common phenomenon [57,68,69,73,74,75].

**Figure 3:**
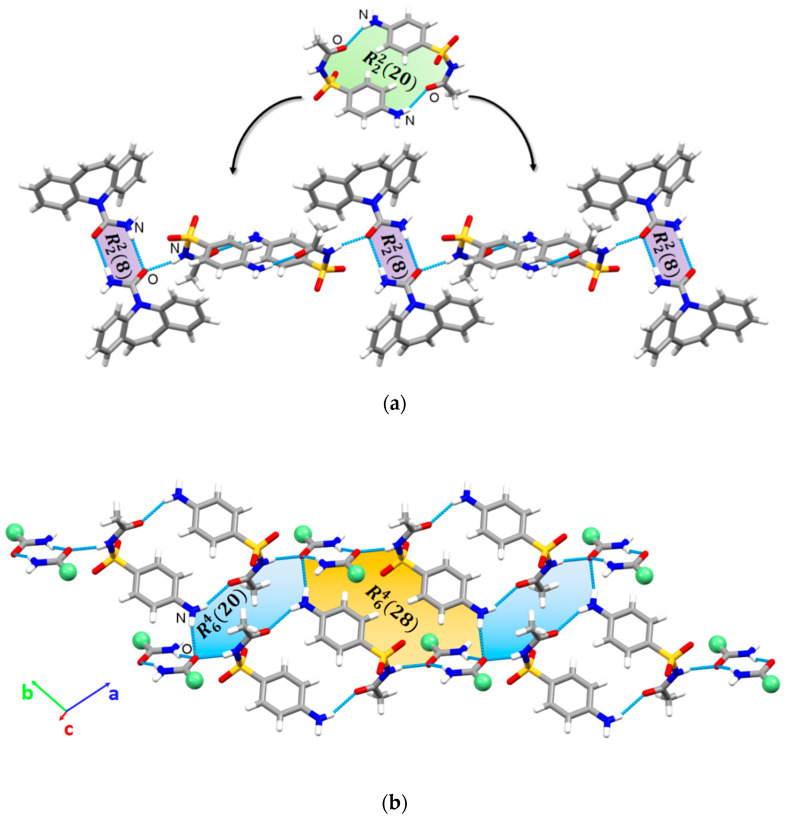
Packing arrangement of the CBZ and SCTM molecules in the [CBZ+SCTM+H_2_O] (1:1:0.7) and [CBZ+SCTM+MeOH] (1:1:0.352) cocrystals: (**a**) a 1D chain of alternately connected CBZ and SCTM homodimers, (**b**) a 2D layer (green balls represent diazepine fragments of the CBZ molecule).

Despite the different nature of the solutes enclosed in the crystal structures, the calculated unit cell similarity index (Π = 0.007 [76]) confirms the isomorphic relationship between the studied multicomponent crystals. In both crystals, the CBZ molecules assemble into typical centrosymmetric homodymers through $R_{2}^{2}(8)$ homosynthons. Analogues to CBZ, the pairs of SCTM molecules are packed as dimeric units via $R_{2}^{2}(20)$ ring motifs. The CBZ and SCTM homodimers are further propagated through N-H···O hydrogen bonds, forming 1D chains (Figure 3a). The neighboring chains are joined into a 2D layer by N-H···O interactions, resulting in $R_{6}^{4}(16)$ and $R_{6}^{4}(28)$ ring motives (Figure 3b). The 2D layered frameworks are not further connected via hydrogen bonds and pack along the a-axis through “inversion cup” stacking contacts between CBZ molecules. In the crystal structure of [CBZ+SCTM+MeOH] (1:1:0.352), the hydroxyl functional group of methanol has been shown to be involved in the formation of $R_{4}^{3}(10)$ cyclic motifs that connect the CBZ amide fragment and the SCTM sulfogroup (Figure S4). Therefore, the solvent molecule participates in the additional binding of the CBZ and SCTM homodimers, which in turn stabilizes the crystal structure of the cocrystal solvate. As for [CBZ+SCTM+H_2_O], its isostructurality to the other crystal would appear to suggest that the water molecules exhibit a comparable arrangement.

### 3.3. Thermal Analysis

**Figure 4:**
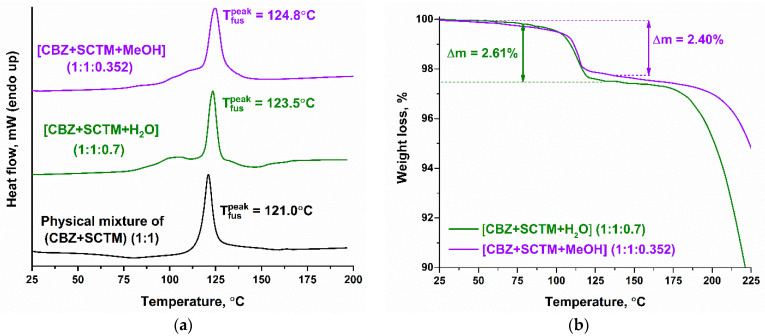
DSC (**a**) and TG (**b**) curves of novel CBZ cocrystal hydrate and cocrystal solvate.

The combination of the DSC and TG techniques was applied to investigate the thermal behavior of the CBZ multicomponent crystals. The resulting thermograms are presented in Figure 4. The DSC curve obtained for an equimolar physical mixture of the parent components showed a single endothermic effect at 121.0 ± 0.2 °C, which corresponds to the melting of the eutectic mixture. The absence of any additional endo- or exo-peaks on the thermogram indicates that cocrystal formation from the eutectic is not occurring [77]. This phenomenon is not an isolated occurrence; a comparable thermal behavior of physical mixtures was previously observed in the CBZ cocrystalline systems with methylparaben [70] or trihydroxybenzoic acids [48]. A salient observation is that had the DSC approach been the sole method employed for screening, all of these systems would have been screened out.

In contrast to the DSC curve of the (CBZ+SCTM) physical mixture, the thermograms for [CBZ+SCNM+H_2_O] (1:1:0.7) and [CBZ+SCTM+MeOH] (1:1:0.352) demonstrate a more complex performance (Figure 4a). In both cases, the DSC curves exhibit two endothermic peaks, of which the first corresponds to the cocrystal dehydration/desolvation, while the second pertains to the melting of the resulting solids following the removal of the solvent from the crystal lattice of multicomponent crystals. Due to the overlap of these processes, the TG experiments were performed. As illustrated in Figure 4b, the TG curves of the cocrystal hydrate and methanol solvate show comparable multistage weight loss processes. The first stage, ranging from 95.8 °C to 119.7 °C for [CBZ+SCTM+H_2_O] or from 103.1 °C to 119.0 °C for [CBZ+SCTM+MeOH], is attributed to the loss of 2.61% or 2.40% of the initial mass, which corresponds to the evaporation of 0.7 moles of water or 0.35 moles of methanol, respectively. The subsequent stages of weight loss are observed at temperatures in excess of 200 °C and are associated with the degradation of the studied samples. In light of the outcomes of the TG experiments, the second endotherm on the DSC curves for the cocrystal hydrate (123.5 ± 0.2 °C) or methanol solvate (124.8 ± 0.2 °C) is not accompanied by any concomitant weight loss.

In order to ascertain the role of the solvent, additional experiments on the dehydration/desolvation of the multicomponent crystals were performed, as has been previously done for the hydrated CBZ cocrystals with trihydroxybenzoic acids [48]. The comparison of the DSC curves and experimental PXRD patterns before and after dehydration/desolvation for [CBZ+SCTM+H_2_O] (1:1:0.7) and [CBZ+SCTM+MeOH] (1:1:0.352) is presented in Figure S5. In accordance with the PXRD patterns, it was determined that the solvent in both multicomponent crystals does not play a structure-forming role, since the crystal structures after the removal of water or methanol remain unchanged. Due to the fact that CBZ is a thermolabile substance [78,79], the synthesis of the anhydrous cocrystal by drying of [CBZ+SCTM+H_2_O] at elevated temperatures is inadvisable.

### 3.4. Design of the Cocrystal Hydrate Preparation Approach

Taking into account the potential of the CBZ cocrystal hydrate from the pharmaceutical point of view, a phase diagram was determined to facilitate the large-scale synthesis of the pure multicomponent crystal, without admixture of the parent components. In the view of the fact that the single crystals of [CBZ+SCTM+H_2_O] (1:1:0.7) were obtained from acetonitrile, this solvent was utilized for the production of the multicomponent crystal by the slurry method.

**Figure 5:**
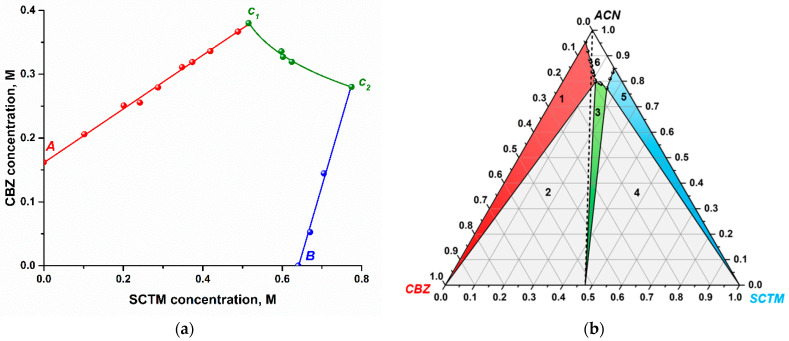
(**a**) Phase solubility diagram of CBZ/SCTM in acetonitrile at 25 °C (Points A and B correspond to the equilibrium solubilities of parent CBZ and SCTM, respectively. Points C1 and C2 indicate the invariant points, at which cocrystal hydrate and CBZ or SCTM coexist at equilibrium); (**b**) ternary phase diagram for the CBZ/SCTM/ACN system at 25 °C in mass fractions (The regions in the diagram are as follows: (1) solid CBZ in equilibrium with the solution, (2) solid CBZ and cocrystal hydrate in equilibrium with the solution, (3) solid cocrystal hydrate in equilibrium with the solution, (4) solid SCTM and cocrystal hydrate in equilibrium with the solution, (5) solid SCTM in equilibrium with the solution, (6) homogeneous solution phase).

The experimental determination of the phase solubility diagram for [CBZ+SCTM+H_2_O] (1:1:0.7) involved four distinct steps (Figure 5a). Firstly, the solubilities of the parent CBZ and SCTM were measured in acetonitrile at 25 °C. It was found that the solubility of SCTM is approximately 4 times that of CBZ. The second and third steps involved measuring the solubility of CBZ as a function of SCTM concentration (red points) and vice versa, solubility of SCTM as a function of CBZ concentration (blue points). Figure 5a demonstrates that the solubility of CBZ or SCTM gradually increases with increasing the concentration of SCTM or CBZ in solution. The observed behavior can be attributed to the weak complexation between the cocrystal components in acetonitrile (Figure S6). At concentrations of the components approximating the equilibrium solubilities of CBZ or SCTM in acetonitrile, two invariant points (*C*_1_ and *C*_2_) were identified, wherein solids of the cocrystal hydrate and one of the parent APIs coexist in equilibrium with the solution. Within the narrow zone between these invariant points, the solubility of CBZ decreases nonlinearly with increasing SCTM concentration (green points).

The resulting phase solubility diagram was utilized to construct a ternary phase diagram for the CBZ/SCTM/ACN system at 25 °C. As demonstrated in Figure 5b, the resulting phase diagram is divided into six regions, of which Regions (1) to (5) correspond to the saturated solutions of differing compositions, while Region (6) represents an undersaturated solution. Regions (1), (3), and (5) denote the solid phases of CBZ (colored in red), [CBZ+SCTM+H_2_O] (1:1:0.7) (colored in green), and SCTM (colored in blue), respectively, which are in equilibrium with the solution. Regions (2) and (4) correspond to the areas wherein two solids, including [CBZ+SCTM+H_2_O] (1:1:0.7) and CBZ or SCTM, coexist in equilibrium with the solution. The experimental PXRD patterns obtained for the residual solids from each region (with the exception of Region (6)) are presented in Figure S7. The experimental concentrations of each component expressed as mass fraction are summarized in Table S1.

Figure 5b shows that the cocrystal hydrate exhibits incongruent dissolution in acetonitrile owing to the fact that Region (3) and the stoichiometric line (dotted line) do not intersect with each other. Moreover, the solid-state region for [CBZ+SCTM+H_2_O] (1:1:0.7) is characterized by a narrow and skewed distribution, with a clear tendency towards the SCTM side. This indicates that if the equimolar (CBZ+SCTM) physical mixture is utilized, the cocrystal hydrate formation will be accompanied by the gradual co-precipitation of less soluble CBZ (Region (2)), as was evidenced in the crystallization experiments. In order to reach Region (3), where the sole stable solid phase is [CBZ+SCTM+H_2_O] (1:1:0.7), it is necessary to use a small excess of SCTM in relation to CBZ.

### 3.5. Effect of pH on Stability and Solubility of Cocrystal Hydrate

Given the inadequate aqueous solubility and dissolution rate of CBZ, it is imperative that the research focuses on investigating the behavior of the novel multicomponent crystal in aqueous medium. This will facilitate a comprehensive understanding and the development of effective delivery mechanisms for the drug. CBZ is recognized as a non-ionizable compound, the solubility of which is unaffected by the pH of an aqueous solution [80]. Moreover, exposure to water instigates the phase transformation of the anhydrous CBZ to its dihydrate form (CBZ·2H_2_O), which exhibits a solubility of (8.30 ± 0.02)·10^−4^ M at 37 °C [81]. In turn, SCTM is an ampholyte (p*K*_a1_ 1.94, p*K*_a2_ 5.26 [82]) with a U-shaped solubility-pH profile. The solubility of SCTM at the isoelectric point (pI 3.6) and 37 °C is (5.10 ± 0.08)·10^−2^ M, which is almost 62 times the solubility of CBZ·2H_2_O. Concurrently, the solubility of SCTM at pH 6.5 exceeds that of CBZ·2H_2_O by more than 1000 times (Figure S8). Considering the significant disparity in solubility of the parent components, it is expected that [CBZ+SCTM+H_2_O] will exhibit low stability and incongruent dissolution, irrespective of the pH of the aqueous solution.

In order to investigate the effect of pH on the stability and solubility of the [CBZ+SCTM+H_2_O] (1:1:0.7) cocrystal, the experiments were performed in buffer solutions with a pH of 3.6 and 6.5. The equilibrium solubility of the cocrystal hydrate as a function of the pH solution was determined at the eutectic point, in accordance with the methodology developed by Good and Rodriguez-Hornedo [83]. For clarity, the eutectic point was achieved by mixing an excess of two solids, including the cocrystal hydrate and the less soluble parent substance (CBZ·2H_2_O), which was further equilibrated in buffer at a temperature of 37 °C. The solid phase composition following equilibration was monitored by PXRD. The results of cocrystal solubility measurements at the eutectic point are summarized in Table 2.

In buffer pH 3.6, wherein the disparity in solubility of the parent compounds is minimal, the eutectic point between [CBZ+SCTM+H_2_O] and CBZ·2H_2_O was reached (Figure S9). The total eutectic concentrations of CBZ and SCTM were used to calculate the eutectic constant (*K*_eu_) and the cocrystal hydrate solubility (*S*_CC_). The *K*_eu_ value, which considerably exceeds the stoichiometric ratio of the studied multicomponent crystal, indicates that [CBZ+SCTM+H_2_O] (1:1:0.7) is thermodynamically unstable but more soluble than the less soluble API. Specifically, the equilibrium solubility of the cocrystal hydrate in the pH 3.6 buffer is 11.2 times that of CBZ·2H_2_O. An increase in the pH value of the dissolution medium from 3.6 to 6.5 rendered it impossible to attain the eutectic point between the two solid phases, owing to a thousand-fold disparity in the solubility of the parent substances. In support of this, Figure S10 demonstrates a series of experimental PXRD patterns of the residual solids obtained by the mixing of excess [CBZ+SCTM+H_2_O] and CBZ·2H_2_O in the buffer solution pH 6.5, wherein the mass of the cocrystal hydrate varied from 100 mg to 200 mg. It was found that despite the gradual increase in the cocrystal hydrate content in the initial mixture, the bottom phase after equilibration was predominantly CBZ·2H_2_O. At higher content of [CBZ+SCTM+H_2_O], co-precipitation of the pure SCTM from the saturated solution was detected. Moreover, the buffering effect of the ampholytic SCTM resulted in a considerable decline in the pH of the dissolution medium in comparison with the initial values (Table 2). It can be hypothesized that the observed behavior of [CBZ+SCTM+H_2_O] is a consequence of the absence of a stable region of the cocrystal hydrate in the pH 6.5 buffer due to its too high solubility [84].

### 3.6. In Vitro Dissolution and Diffusion Tests

**Figure 6:**
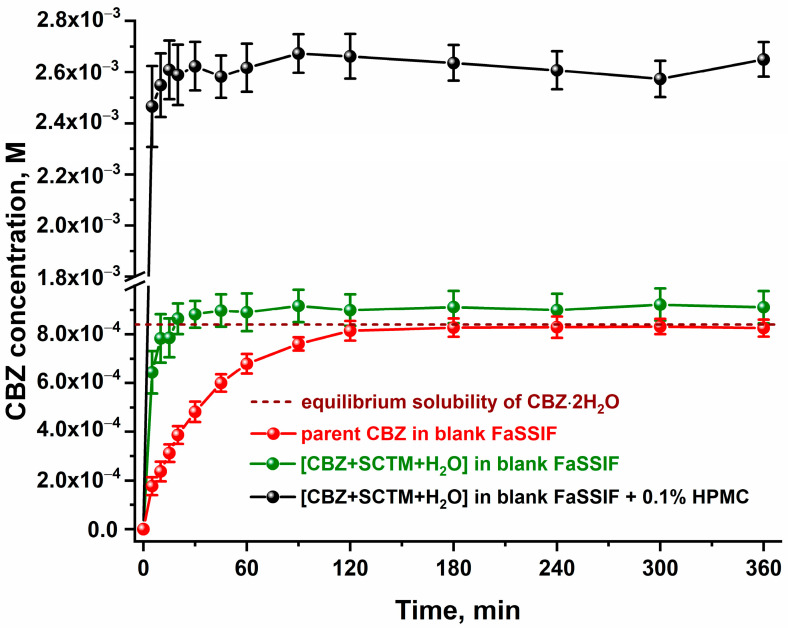
The powder dissolution profiles of the parent CBZ and [CBZ+SCTM+H_2_O] (1:1:0.7) in blank FaSSIF in the absence or presence of pre-dissolved HPMC at 37 °C.

The next stage of our study involved the investigation of the dissolution and diffusion performance of the highly soluble cocrystal hydrate in relation to the parent CBZ. The dissolution profiles for the parent CBZ and [CBZ+SCTM+H_2_O] (1:1:0.7) in blank FaSSIF (pH 6.5) are shown in Figure 6.

As illustrated in Figure 6, a poorly soluble CBZ exhibits a reduced dissolution rate. Consequently, a concentration level equal to the equilibrium solubility of CBZ·2H_2_O is only attained after a duration of 180 min of the dissolution experiment. In contrast, [CBZ+SCTM+H_2_O] demonstrates an enhanced dissolution behavior, requiring a mere 20 min to reach the same level of CBZ concentration in an aqueous solution as the parent API. Due to the extremely low thermodynamic stability of the cocrystal hydrate in the buffer solution, [CBZ+SCTM+H_2_O] undergoes a rapid dissociation into its components upon contact with the dissolution medium (Figure S11a). This, in turn, initiates the quick conversion of the anhydrous CBZ to its dihydrate form in comparison to the dissolution behavior of the parent API.

In an attempt to prevent the rapid solution-mediated phase transformation of [CBZ+SCTM+H_2_O], the dissolution experiment for the multicomponent crystal was additionally performed in the buffer solution containing a pre-dissolved HPMC. The efficacy of this polymer has previously been demonstrated on a number of CBZ cocrystals [48,70]. Figure 6 demonstrates that the use of HPMC during the cocrystal hydrate dissolution results in the generation of a stable supersaturation state of CBZ in solution over 360 min. Specifically, the CBZ concentration in this state exceeds the CBZ·2H_2_O solubility by 3.2 times. Despite an increase in the CBZ concentration, it was found that HPMC did not contribute to the stabilization of the cocrystal hydrate in buffer solution, which is still characterized by incongruent dissolution (Figure S11b). The PXRD analysis of the residual solids confirmed that all of the tested samples converted into CBZ·2H_2_O (Figure S12).

**Figure 7:**
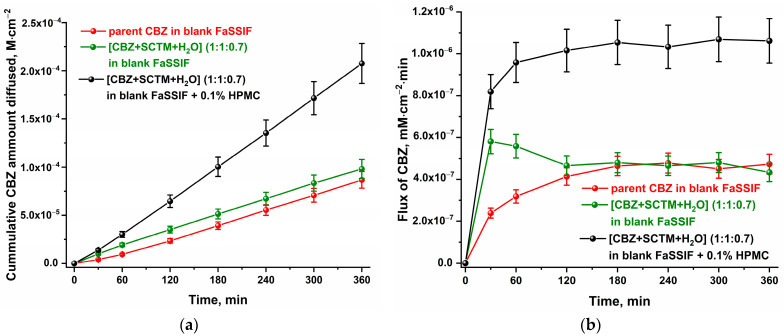
The plots of (**a**) cumulative amount diffused and (**b**) flux vs. time for [CBZ+SCTM+H_2_O] (1:1:0.7) in comparison with parent CBZ.

Considering the effect of cocrystallization on the CBZ dissolution rate, the diffusion behavior of [CBZ+SCTM+H_2_O] was also determined. The resulting plots of the cumulative amount of CBZ diffused and flux are shown in Figure 7. A comparison of the plots for the parent CBZ and [CBZ+SCTM+H_2_O] in blank FaSSIF demonstrated that, despite comparable profiles of the cumulative amount diffused of API, the cocrystal hydrate exhibits improved flux over the first 180 min due to its higher dissolution rate. Conversely, the presence of pre-dissolved HPMC in the dissolution medium resulted in a significant enhancement of the CBZ diffusion performance in [CBZ+SCTM+H_2_O]. CBZ supersaturation in the donor chamber is reflected in the rapid increase in CBZ concentration in the receptor chamber, which is itself a consequence of the concentration gradient between the two chambers. As a result, the cumulative CBZ amount diffused through the membrane is 2.4 times higher than that for parent API.

### 3.7. In Vivo Pharmacokinetic Study

**Figure 8:**
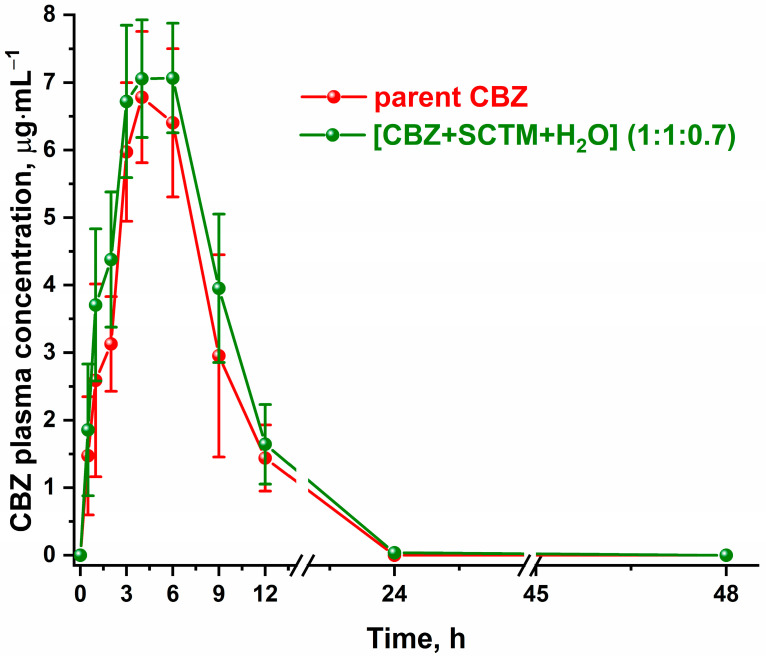
Pharmacokinetic profiles of the parent CBZ and [CBZ+SCTM+H_2_O] (1:1:0.7) after oral dosing to rabbits.

In order to provide a comprehensive evaluation of the novel cocrystal hydrate on the CBZ bioavailability, in vivo experiments were performed. The mean plasma concentration–time curves for the parent CBZ and [CBZ+SCTM+H_2_O] (1:1:0.7) in rabbits are shown in Figure 8. The pharmacokinetic parameters, including maximal concentration of CBZ (*C*_max_), time to maximal concentration (*t*_max_), and area under the concentration–time curve (AUC_0–∞_), are summarized in Table 3.

As illustrated in Figure 8, the pharmacokinetic profiles of the parent CBZ and [CBZ+SCTM+H_2_O] exhibited a comparable shape with a single absorption peak. This aligns with the observation that all three pharmacokinetic parameters in Table 3 are also closely similar for both tested samples. The primary cause of the observed behavior of [CBZ+SCTM+H_2_O] has been ascribed to its extremely low thermodynamic stability. Concurrently, it was established that the maximum CBZ concentration in blood is attained at a marginally accelerated rate for the cocrystal hydrate, which is characterized by its higher dissolution rate in comparison to the pure API. In summary, the present study demonstrates that the novel CBZ multicomponent crystal displays comparable bioavailability to the parent API and can serve as alternative solid dosage forms for carbamazepine, exhibiting a combined pharmacological effect.

## 4. Conclusions

The present study presents the results of an investigation of two novel drug–drug cocrystalline forms of carbamazepine with sulfacetamide, identified during liquid-assisted grinding. In accordance with the experimental data from the results of single-crystal X-ray diffraction and thermal analysis, the multicomponent solids were classified as cocrystal hydrate and cocrystal methanol solvate. Despite the incorporation of different solvent molecules in the crystal structures of novel cocrystals, the comparable crystallographic cell parameters and packing modes revealed the isostructural nature of these solids. Due to additional dehydration/desolvation experiments, it was determined that the solvent molecules within the studied crystal structures serve a stabilizing function rather than a structure-forming one.

In order to optimize the large-scale synthesis of the pure cocrystal hydrate on large scale, a ternary phase diagram for the carbamazepine-sulfacetamide-acetonitrile system was constructed. It was found that the cocrystal hydrate has a narrow thermodynamically stable region, shifted towards the side of sulfacetamide. In aqueous media, the ampholytic nature of sulfacetamide exerts a substantial impact on the cocrystal hydrate stability and solubility in relation to pH. The low stability of the cocrystal hydrate in water resulted in comparable in vitro and in vivo characteristics of carbamazepine in comparison to the parent drug substance.

## Figures and Tables

**Table 1:** Crystallographic data for the novel CBZ cocrystal hydrate and methanol solvate.

	[CBZ+SCTM+H_2_O] (1:1:0.7) ^a^	[CBZ+SCTM+MeOH] (1:1:0.352)
Chemical formula	C_15_H_12_N_2_O·C_8_H_10_N_2_O_3_S	C_15_H_12_N_2_O·C_8_H_10_N_2_O_3_S·0.352(CH_4_O)
*M*_r_	450.50	461.73
Crystal system, space group	Triclinic, *P*-1	Triclinic, *P*-1
Temperature, K	120	100
*a*, Å	10.0803 (5)	10.0837 (4)
*b*, Å	10.2836 (6)	10.1781 (4)
*c*, Å	11.8893 (7)	11.7782 (5)
α, °	106.1941 (19)	106.2160 (13)
β, °	99.903 (2)	99.5884 (14)
γ, °	95.703 (2)	95.9646 (14)
*V* (Å^3^)	1151.70 (11)	1129.98 (8)
*Z*	2	2
*D*_calc_, g·cm^−3^	1.299	1.357
*μ*, mm^−1^	0.18	0.18
Reflection collected	18,645	19,269
Independent reflections	5011	6905
Reflections with *I* > 2(*I*)	4357	5620
*R*_int_	0.032	0.031
*R*_1_[*F*^2^ > 2σ(*F*^2^)], *wR*_2_(*F*^2^), *S*	0.0544, 0.1211, 1.097	0.0432, 0.1108, 1.042
Parameters	377	431
Largest diff. peak/hole, e·Å^−3^	0.33, −0.47	0.38, −0.53
CCDC	2442631	2442630

^a^ The disorder water molecules were removed by the SQUEEZE procedure of the program PLATON.

**Table 2:** The values of initial and final (pH_eq_) pH of buffer solution, solid-state composition of residual solid, eutectic constant, and equilibrium solubility of the cocrystal hydrate in aqueous medium at 37 °C.

pH_initial_	pH_eq_	Solids in Equilibrium with the Solution	*K*_eu_	*S*_CC_, M
3.6	3.6	[CBZ+SCTM+H_2_O], CBZ·2H_2_O	26.4 ± 0.5	(9.32 ± 0.15)·10^−3^
6.5	4.5	CBZ·2H_2_O	-	-
6.5	4.5	CBZ·2H_2_O, SCTM	-	-
6.5	4.4	CBZ·2H_2_O, SCTM	-	-

**Table 3:** Main pharmacokinetic parameters of the parent CBZ and [CBZ+SCTM+H_2_O] (1:1:0.7) after oral administration to rabbits.

	CBZ *	[CBZ+SCTM+H_2_O] (1:1:0.7)
*C*_max_, μg·mL^−1^	7.7 ± 1.1	7.8 ± 0.5
*t*_max_, h	5.3 ± 1.2	4.7 ± 1.2
AUC_0–∞_, μg·h·mL^−1^	58 ± 9	68 ± 11

* data are taken from [48].

## Data Availability

The results obtained for all experiments performed are shown in the manuscript and Supplementary Materials, the raw data will be provided upon request.

## Supplementary Materials

The following supporting information can be downloaded at: https://www.mdpi.com/article/10.3390/pharmaceutics17050678/s1, Figure S1: Comparison of the experimental PXRD patterns of the ground (CBZ+SCTM) (1:1) samples in the presence of water or acetone with the calculated PXRD patterns for CBZ dihydrate or acetone solvate; Figure S2: Comparison of the experimental PXRD patterns of the ground (CBZ+SCTM) (1:1) samples in the presence of acetonitrile, methanol or ethanol with the calculated PXRD patterns of the CBZ polymorphs; Figure S3: The calculated PXRD patterns for the cocrystal hydrate and methanol solvate; Figure S4: Hydrogen-bonded motif for [CBZ+SCTM+MeOH] (1:1:0.352), wherein the purple balls represent hydrocarbon radical of methanol; Figure S5: Comparison of the DSC curves and experimental PXRD patterns before and after dehydration/desolvation of (a) [CBZ+SCTM+H_2_O] (1:1:0.7) and (b) [CBZ+SCTM+MeOH] (1:1:0.352); Figure S6: Solubility dependences of (a) CBZ as a function of SCTM concentration and (b) SCTM as a function of CBZ concentration in acetonitrile at 25 °C; Figure S7: The experimental PXRD patterns of the residual solids obtained from the different regions of the ternary phase diagram in acetonitrile: (a) Region 1, (b) Region 2, (c) Region 3, (d) Region 4, and (e) Region 5; Figure S8: Solubility-pH profile of SCTM; Figure S9: Comparison of the experimental PXRD pattern of the solid after solubility experiment in the buffer solution pH 3.6 confirming the simultaneous presence of the cocrystal hydrate and CBZ dihydrate in equilibrium; Figure S10: Comparison of the experimental PXRD patterns of the residual solids obtained by mixing excess [CBZ+SCTM+H_2_O] (1:1:0.7) and CBZ∙2H_2_O in the buffer solution pH 6.5 at 37 °C; Figure S11: The concentration–time profiles of CBZ and SCTM obtained as a result of the [CBZ+SCTM+H_2_O] (1:1:0.7) dissolution in blank FaSSIF in the (a) absence or (b) presence of the pre-dissolved HPMC (0.1% *w*/*v*) at 37 °C; Figure S12: The experimental PXRD patterns of residual solids after the cocrystal hydrate dissolution experiments; Table S1: Composition of solid phases and mass fractions of CBZ, SCTM and ACN used for construction of the ternary phase diagram at 25 °C.
