# Supplementary material for: Novel Drug–Drug Cocrystalline Forms of Carbamazepine with Sulfacetamide: Preparation, Characterization, and In Vitro/In Vivo Performance Evaluation
Source: Pharmaceutics. 2025 May 21;17(5):678. doi: 10.3390/pharmaceutics17050678 (PMC12115326; doi:10.3390/pharmaceutics17050678)
Supplement: Supplementary file 1 [file pharmaceutics-17-00678-s001.zip › pharmaceutics-3622362-supplementary.pdf]

Supporting Information

for

Novel drug-drug cocrystalline forms of carbamazepine with sulfacetamide: preparation, characterization and in vitro/in vivo performance evaluation

Denis E. Boycov,[a] *Ksenia V. Drozd,*[a] *Alex N. Manin,*[a] *Andrei V. Churakov,*[b] *Mikhail Yu. Vlasov,*[c] *Irina V. Kachalkina* [c] *and German L. Perlovich*[a*]

[a] G.A. Krestov Institute of Solution Chemistry of the Russian Academy of Sciences, 1 Akademicheskaya St., Ivanovo, 153045, Russian Federation

[b] Institute of General and Inorganic Chemistry of the Russian Academy of Sciences, 31 Leninsky Prosp., Moscow, 119991, Russian Federation

[c] Samara State Medical University of the Ministry of Health of the Russian Federation, 89 Chapayevskaya St., Samara, 443099, Russian Federation

[*]Corresponding author: glp@isc-ras.ru

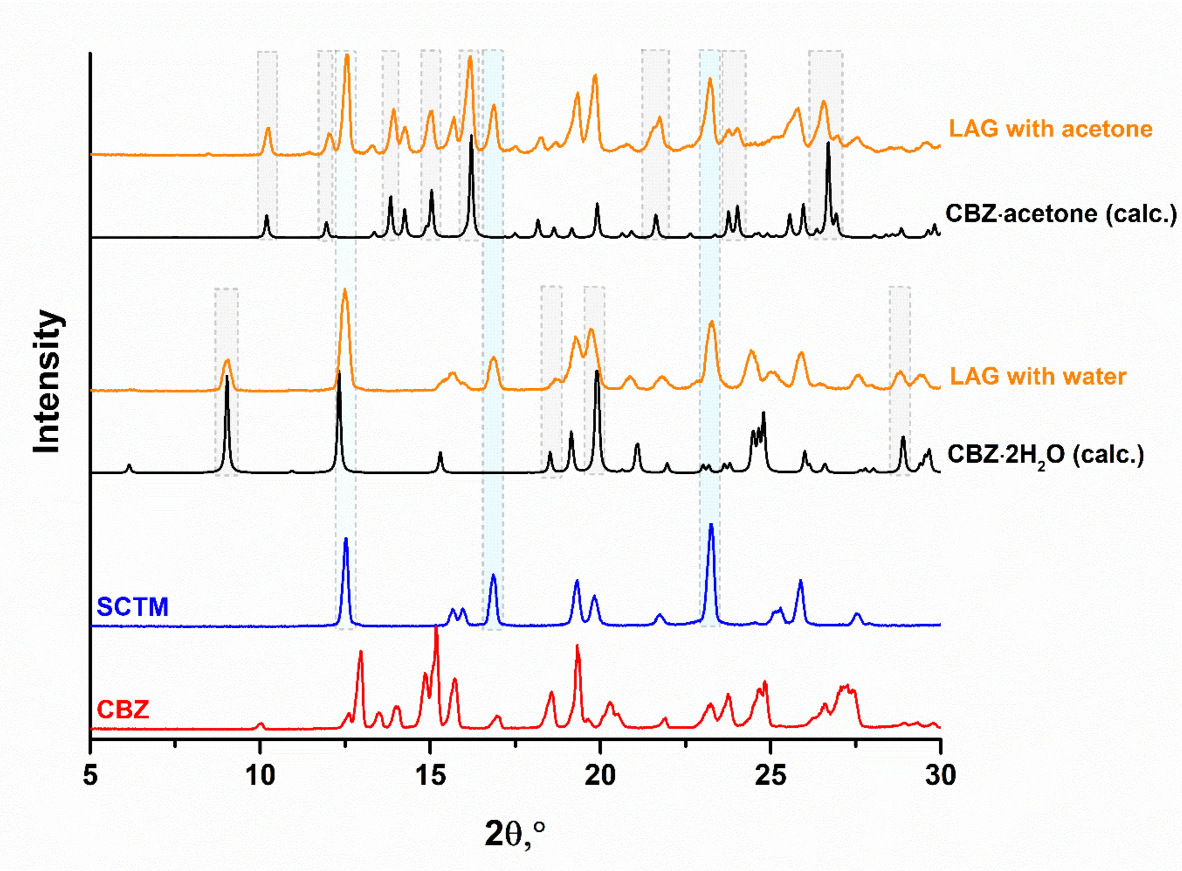

Figure S1. Comparison of the experimental PXRD patterns of the ground (CBZ+SCTM) (1:1) samples in the presence of water or acetone with the calculated PXRD patterns for CBZ dihydrate or acetone solvate.

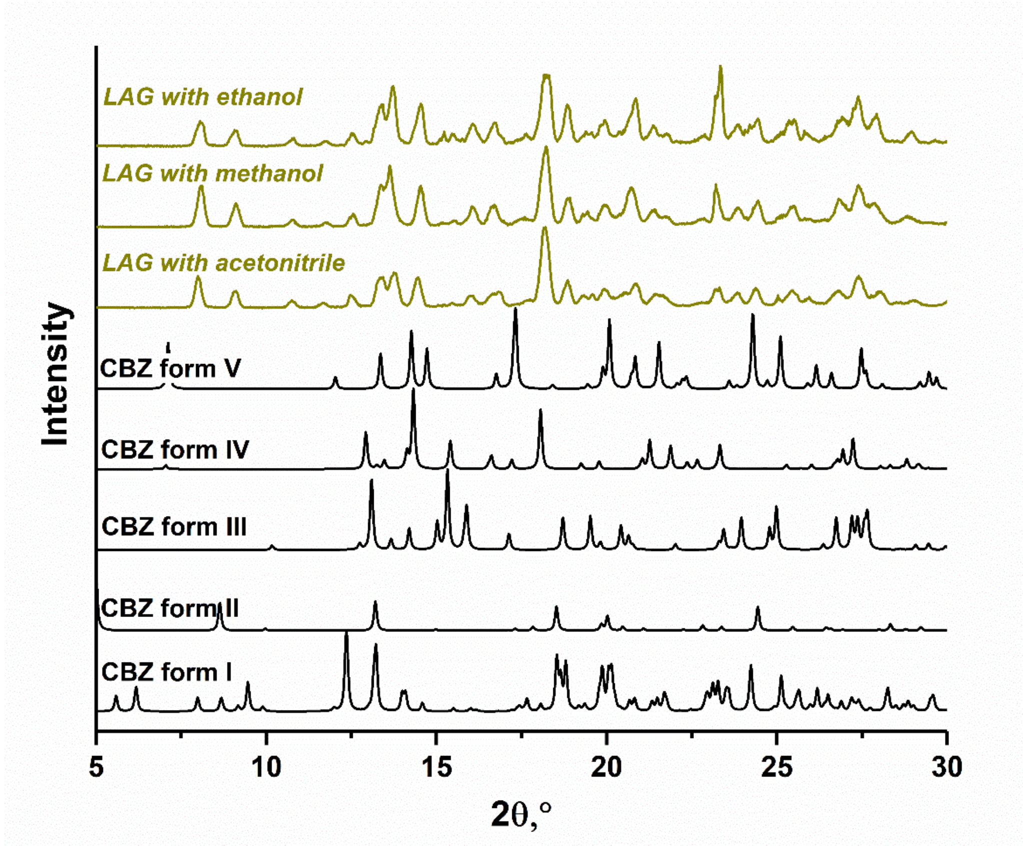

Figure S2. Comparison of the experimental PXRD patterns of the ground (CBZ+SCTM) (1:1) samples in the presence of acetonitrile, methanol or ethanol with the calculated PXRD patterns of the CBZ polymorphs.

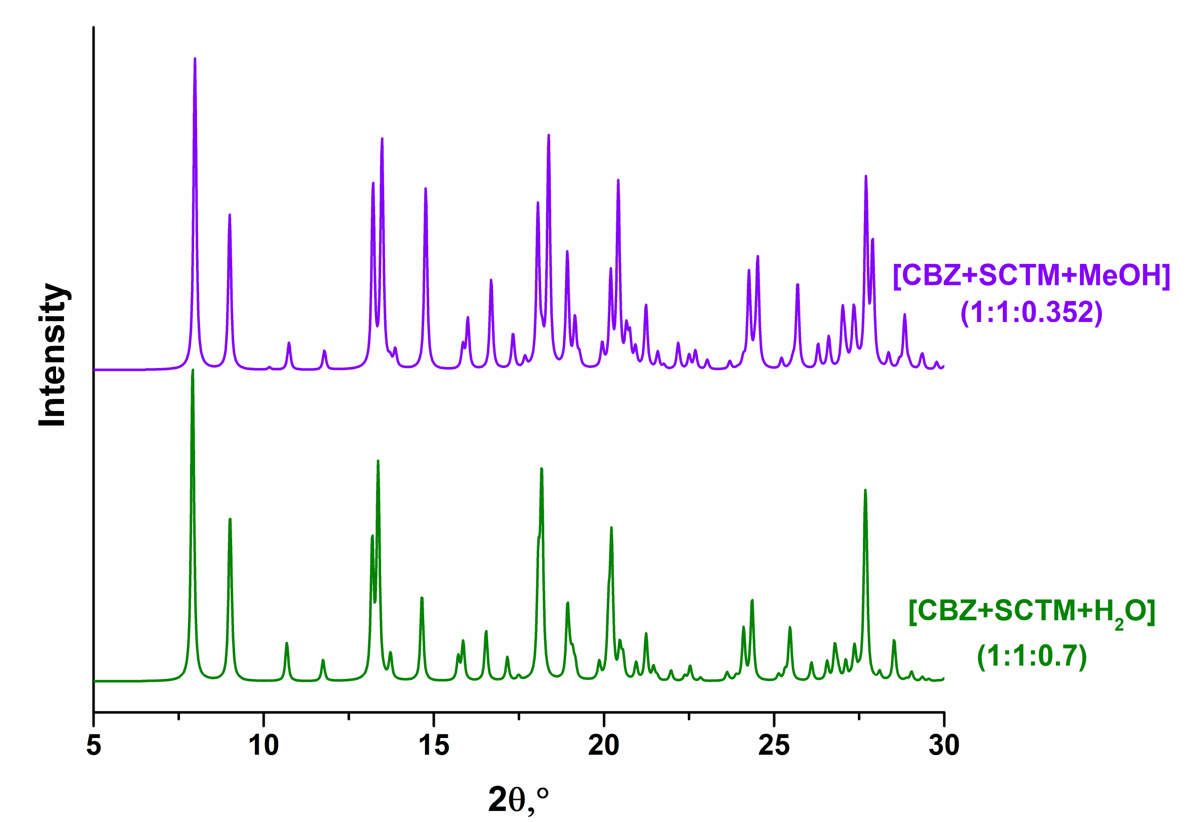

Figure S3. The calculated PXRD patterns for the cocrystal hydrate and methanol solvate.

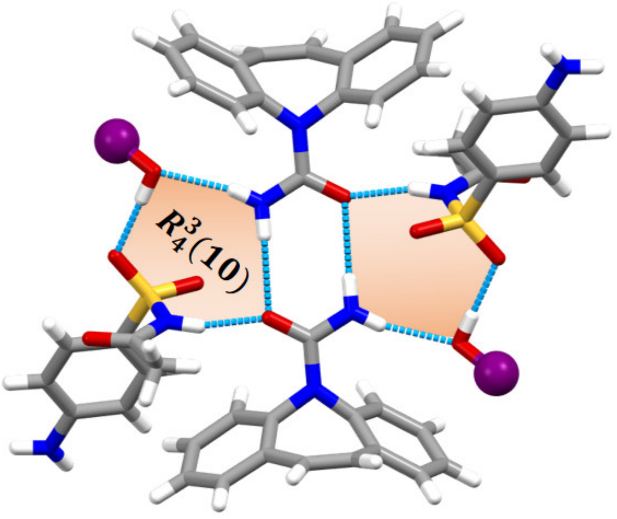

Figure S4. Hydrogen-bonded motif for [CBZ+SCTM+MeOH] (1:1:0.352), wherein the purple balls represent hydrocarbon radical of methanol.

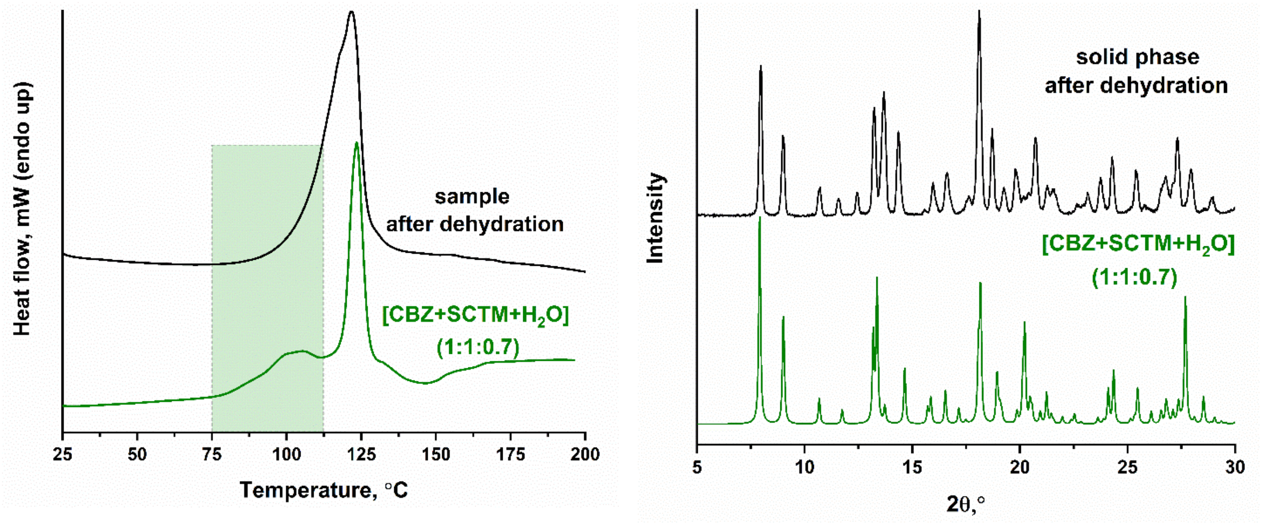

(a)

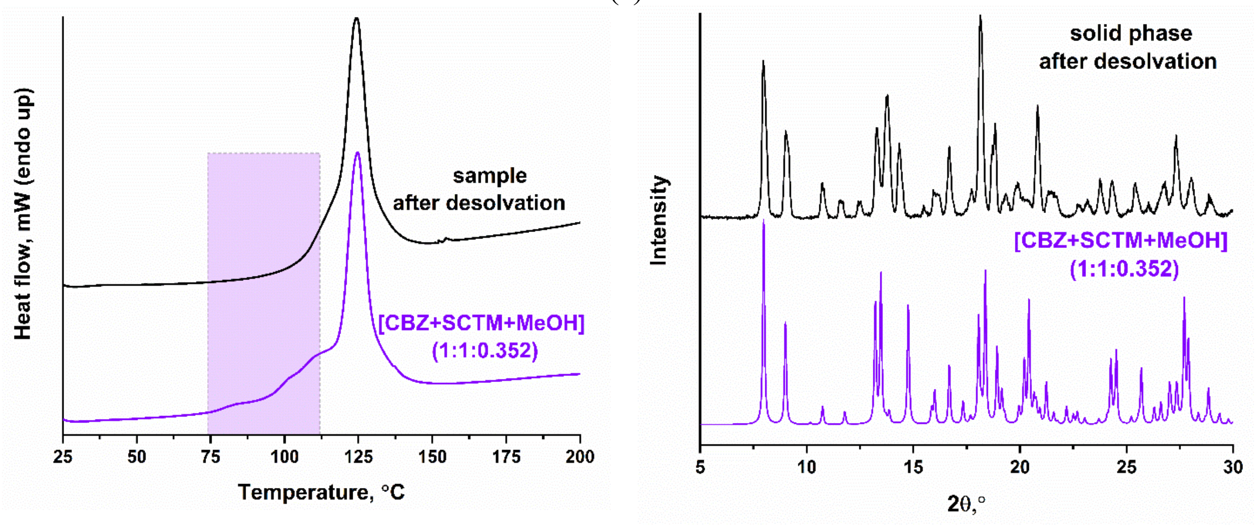

(b)

Figure S5. Comparison of the DSC curves and experimental PXRD patterns before and after dehydration/desolvation of (a) [CBZ+SCTM+H_2O] (1:1:0.7) and (b) [CBZ+SCTM+MeOH] (1:1:0.352).

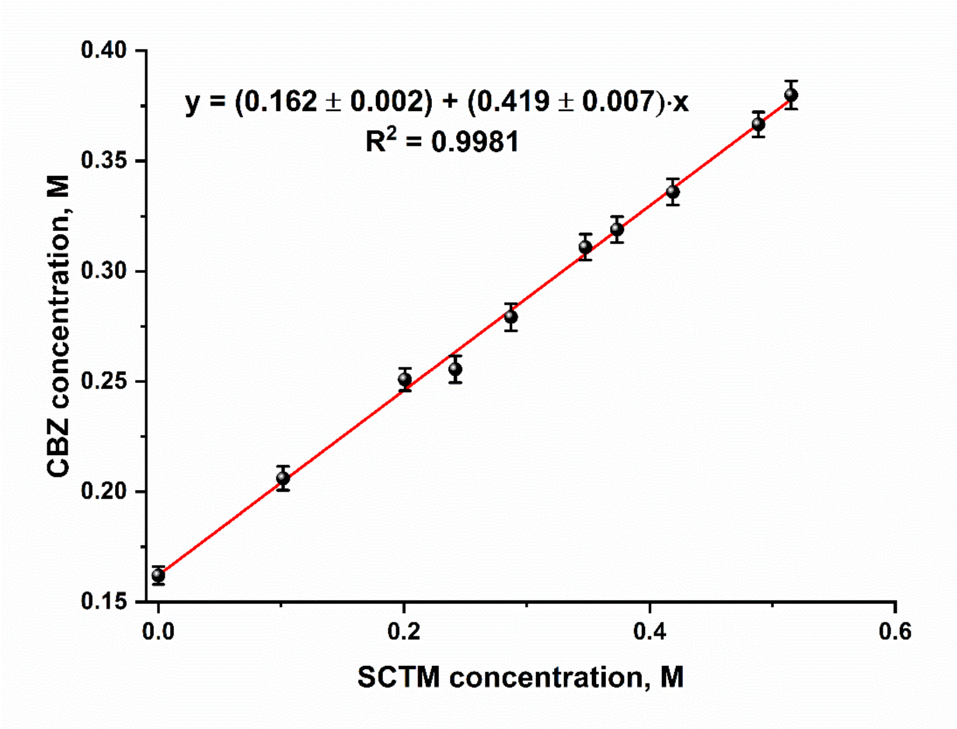

(a)

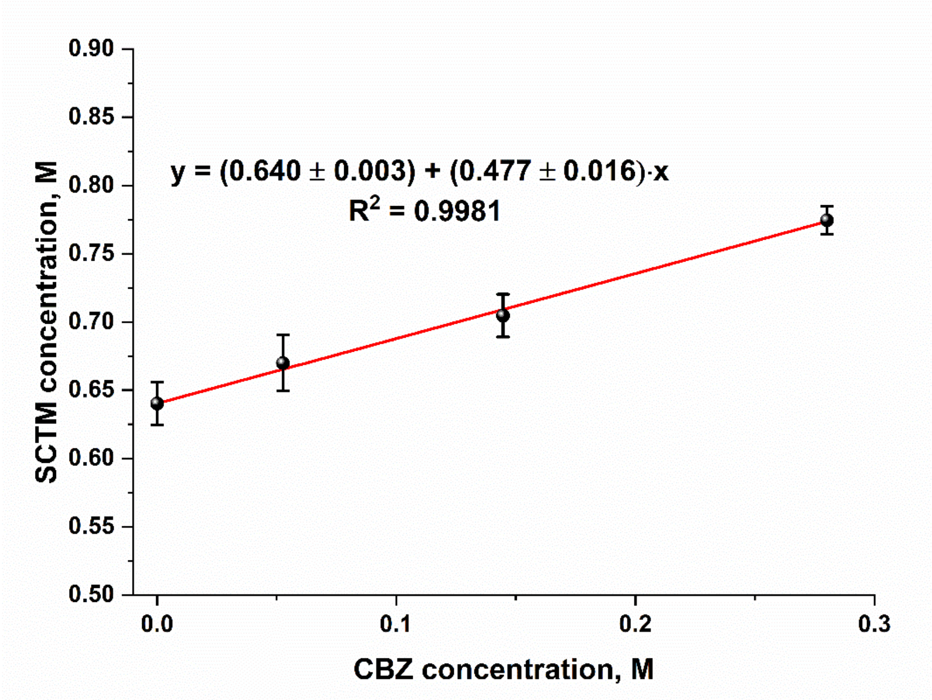

(b)

Figure S6. Solubility dependences of (a) CBZ as a function of SCTM concentration and (b) SCTM as a function of CBZ concentration in acetonitrile at 25°C.

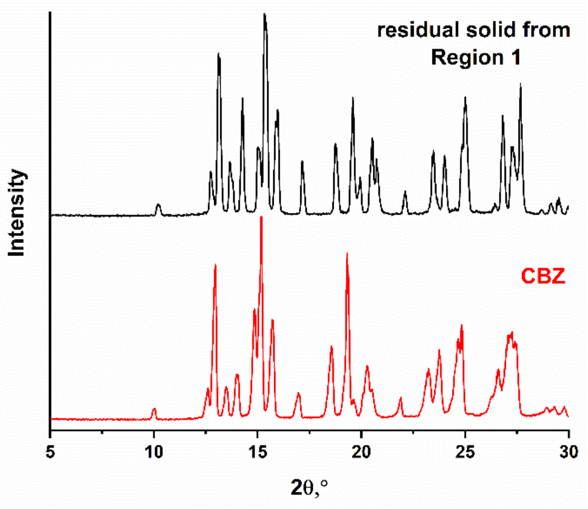

(a)

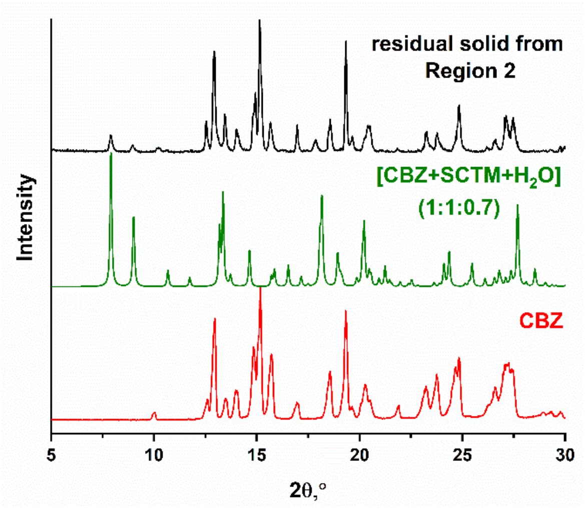

(b)

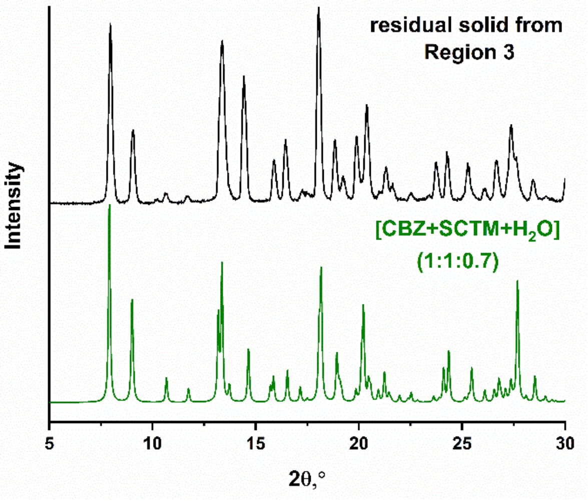

(c)

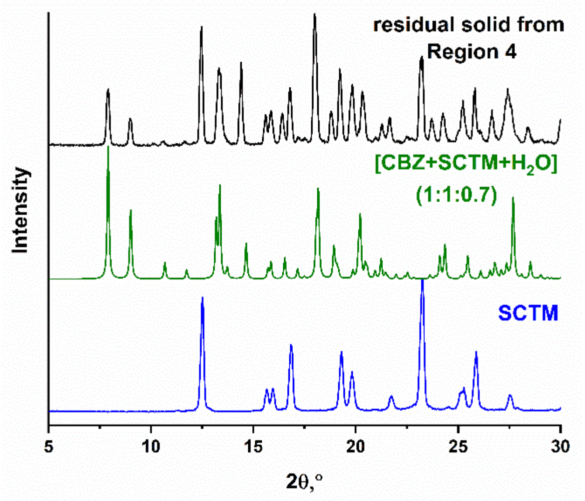

(d)

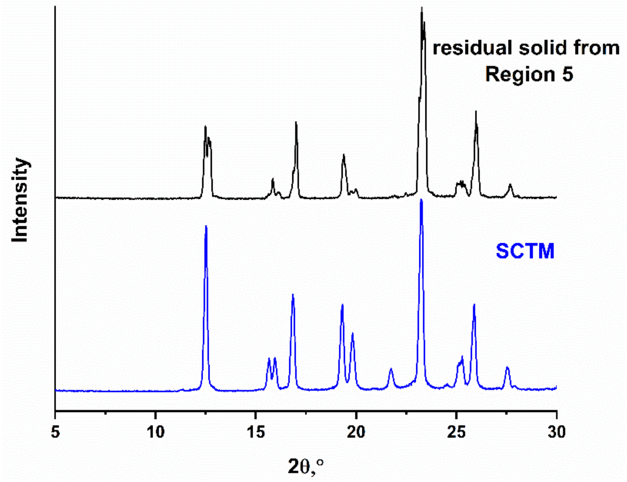

(e)

Figure S7. The experimental PXRD patterns of the residual solids obtained from the different regions of the ternary phase diagram in acetonitrile: (a) Region 1, (b) Region 2, (c) Region 3, (d) Region 4 and (e) Region 5.

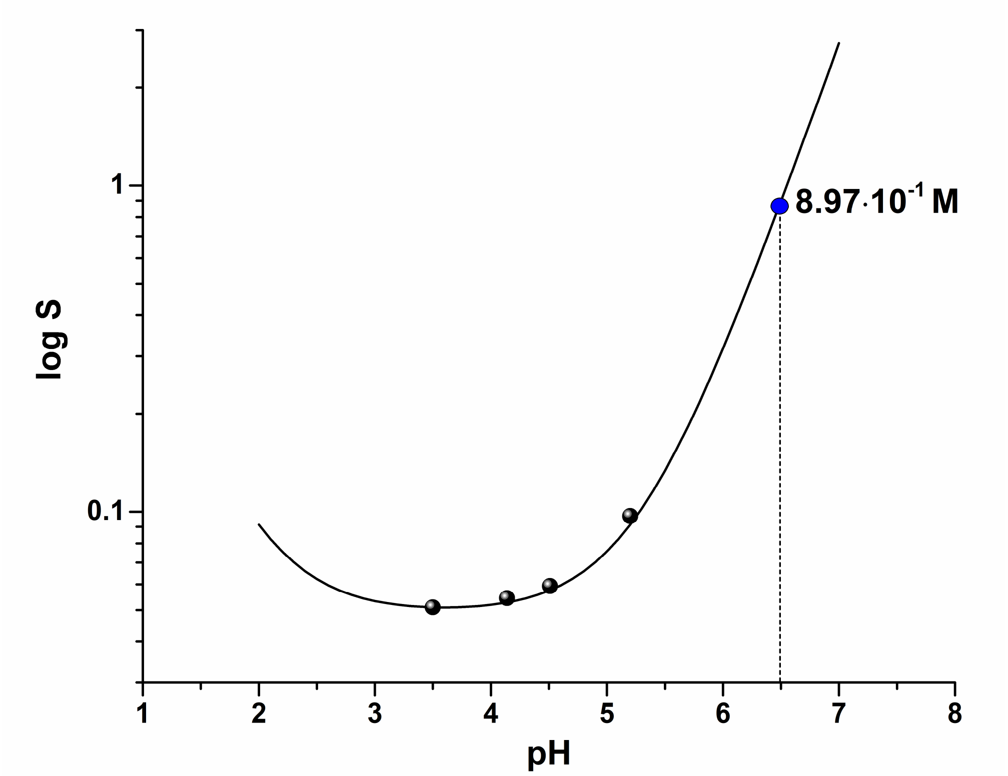

Figure S8. Solubility-pH profile of SCTM. The black balls represent experimental solubilities of SCTM at 37°C: $(5.10\pm0.08)\cdot10^{-2}$ M a pH 3.6, $(5.44\pm0.06)\cdot10^{-2}$ M a pH 4.14, $(5.96\pm0.12)\cdot10^{-2}$ M a pH 4.5 and $(9.70\pm0.08)\cdot10^{-2}$ M a pH 5.2. pH values correspond to equilibrium pH. The experimental values were fitted with Henderson-Hasselbalch equation. The blue circle correspond to the calculated solubility value of SCTM at pH 6.5.

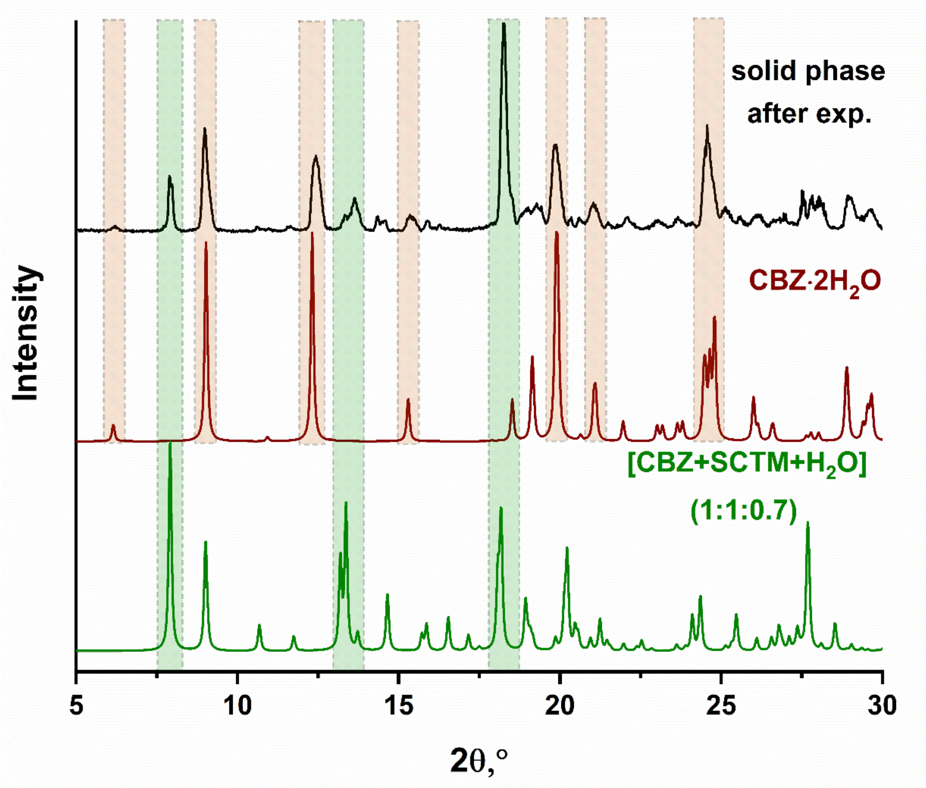

Figure S9. Comparison of the experimental PXRD pattern of the solid after solubility experiment in the buffer solution pH 3.6 confirming the simultaneous presence of the cocrystal hydrate and CBZ dihydrate in equilibrium.

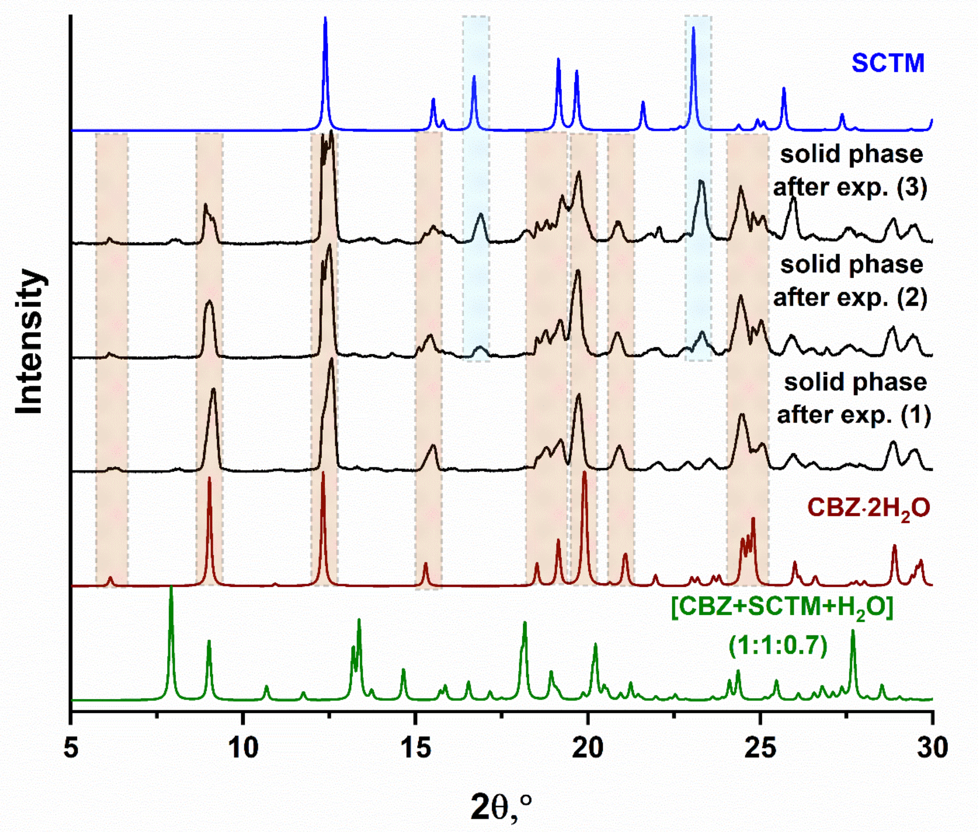

Figure S10. Comparison of the experimental PXRD patterns of the residual solids obtained by mixing excess [CBZ+SCTM+H_2O] (1:1:0.7) and CBZ·2H_2O in the buffer solution pH 6.5 at 37°C. The mass of [CBZ+SCTM+H_2O] (1:1:0.7) was 100 mg, 120 mg and 200 mg.

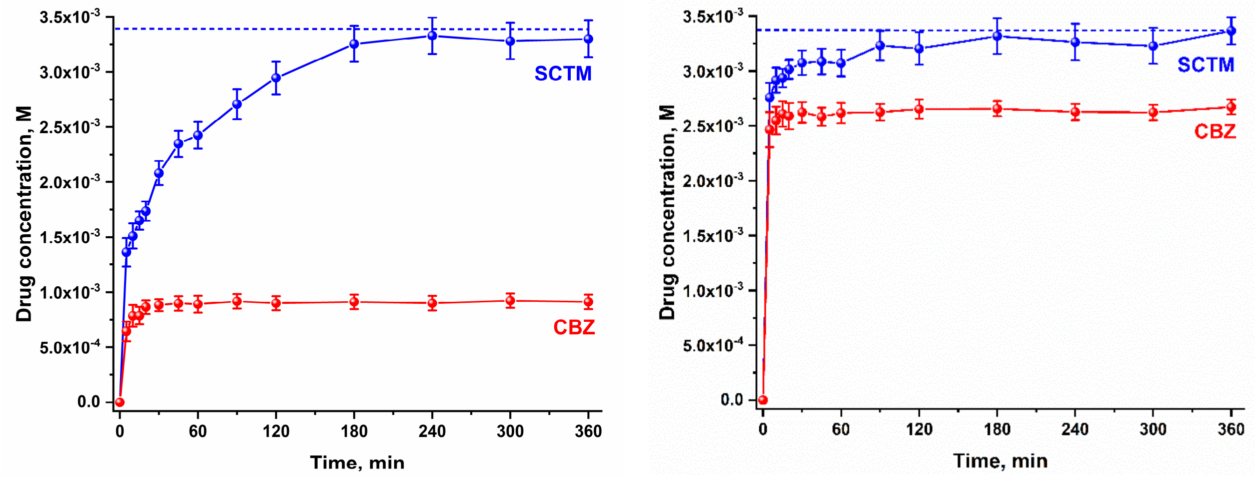

(a) (b)

Figure S11. The concentration-time profiles of CBZ and SCTM obtained as a result of the [CBZ+SCTM+H_2O] (1:1:0.7) dissolution in blank FaSSIF in the (a) absence or (b) presence of the pre-dissolved HPMC (0.1% w/v) at 37°C. The dotted blue line indicates the maximum concentration of SCTM during the cocrystal hydrate dissolution.

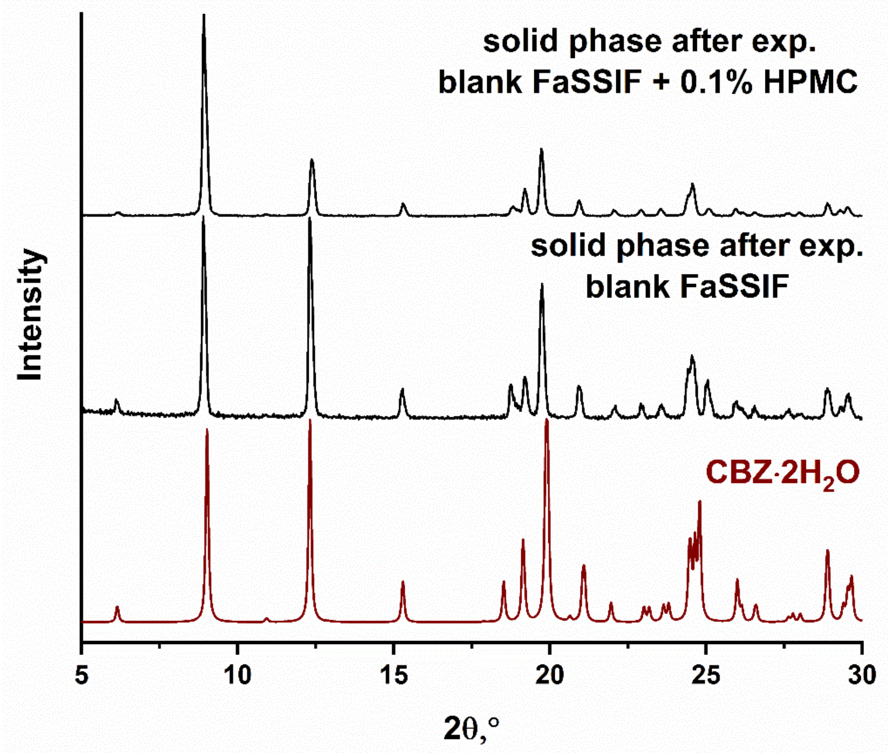

Figure S12. The experimental PXRD patterns of residual solids after the cocrystal hydrate dissolution experiments.

Table S1. Composition of solid phases and mass fractions of CBZ, SCTM and ACN used for construction of the ternary phase diagram at 25°C

Solid phase at equilibrium	CBZ	SCTM	ACN
CBZ	$4.64\cdot10^{-2}$	0	$9.54\cdot10^{-1}$
CBZ	$5.68\cdot10^{-2}$	$2.54\cdot10^{-2}$	$9.18\cdot10^{-1}$
CBZ	$6.67\cdot10^{-2}$	$4.84\cdot10^{-2}$	$8.85\cdot10^{-1}$
CBZ	$6.72\cdot10^{-2}$	$5.76\cdot10^{-2}$	$8.75\cdot10^{-1}$
CBZ	$7.22\cdot10^{-2}$	$6.73\cdot10^{-2}$	$8.61\cdot10^{-1}$
CBZ	$7.86\cdot10^{-2}$	$7.97\cdot10^{-2}$	$8.42\cdot10^{-1}$
CBZ	$8.00\cdot10^{-2}$	$8.49\cdot10^{-2}$	$8.35\cdot10^{-1}$
CBZ	$8.30\cdot10^{-2}$	$9.39\cdot10^{-2}$	$8.23\cdot10^{-1}$
CBZ	$8.85\cdot10^{-2}$	$1.07\cdot10^{-1}$	$8.04\cdot10^{-1}$
CBZ and [CBZ+SCTM+H_2O]	$9.09\cdot10^{-2}$	$1.12\cdot10^{-1}$	$7.97\cdot10^{-1}$
[CBZ+SCTM+H_2O]	$7.98\cdot10^{-2}$	$1.29\cdot10^{-1}$	$7.91\cdot10^{-1}$
[CBZ+SCTM+H_2O]	$7.78\cdot10^{-2}$	$1.30\cdot10^{-1}$	$7.92\cdot10^{-1}$
[CBZ+SCTM+H_2O]	$7.58\cdot10^{-2}$	$1.34\cdot10^{-1}$	$7.90\cdot10^{-1}$
SCTM and [CBZ+SCTM+H_2O]	$6.49\cdot10^{-2}$	$1.63\cdot10^{-1}$	$7.72\cdot10^{-1}$
SCTM	$3.52\cdot10^{-2}$	$1.55\cdot10^{-1}$	$8.10\cdot10^{-1}$
SCTM	$1.32\cdot10^{-2}$	$1.52\cdot10^{-1}$	$8.35\cdot10^{-1}$
SCTM	0	$1.49\cdot10^{-1}$	$8.51\cdot10^{-1}$
